# Supplementary material for: Deep learning models for unbiased sequence-based PPI prediction plateau at an accuracy of 0.65
Source: Bioinformatics. 2025 Jul 15;41(Suppl 1):i590–8. doi: 10.1093/bioinformatics/btaf192 (PMC12261406; doi:10.1093/bioinformatics/btaf192)
Supplement: btaf192_Supplementary_Data [file btaf192_supplementary_data.zip › btaf192_Supplementary_Data/Bernett.79.ALTTEXT.pdf]

Alt-text for the figures

March 26, 2025

I did not know how to supply alt-text in the document. Here is my alt-text for all the figures.

- Fig 1: Graphical abstract summarizing our analyses in six subpanels (see caption).
- Fig 2: Line plot comparing changes in accuracy for nine different tested models for different sizes of the ESM-2 embedding. All models except RFC-40 and Richoux-like perform worse with larger embeddings, those two perform best with t36. Generally, t48 also performs worse than t36.
- Fig 3: Line plot comparing changes in accuracy for four different models for adding a transformer encoder to the architecture. Only for Richoux-like, this results in a considerable performance decrease.
- Fig 4: Line plot comparing changes in accuracy for six models for removing the spectral normalization. For all models except Richoux-ESM-2, this results in random performance.
- Fig 5: Line plot comparing changes in accuracy for three models for placing the encoder at different positions. For all models, placing it after the dimensionality-reducing layers boosts performance.
- Fig 6: Two heatmaps, one showing the real distance map colored by Angstrom, one showing the implicitly predicted distance map (values of the penultimate layer to the model). The Pearson Correlation between both matrices is 0.05. Visually, there is also no similarity.
