# Supplementary material for: Deep learning models for unbiased sequence-based PPI prediction plateau at an accuracy of 0.65
Source: Bioinformatics. 2025 Jul 15;41(Suppl 1):i590–8. doi: 10.1093/bioinformatics/btaf192 (PMC12261406; doi:10.1093/bioinformatics/btaf192)
Supplement: btaf192_Supplementary_Data [file btaf192_supplementary_data.zip › btaf192_Supplementary_Data/Bernett.79.sup.1.pdf]

Deep learning models for unbiased sequence-based PPI prediction plateau at an accuracy of 0.65

Supplementary information

1 Supplementary methods

1.1 Baseline embeddings

In order to investigate the relative gain of the ESM-2 embeddings over other input representations, we tested all architectures with the embeddings of Bepler and Berger [2019], a smaller language model used by D-SCRIPT [Sledzieski et al., 2021]. Further, the model by Richoux et al. [2019] uses one-hot encoding in its original implementation. We also tested all models with this encoding to obtain a baseline embedding performance.

Bepler and Berger embedding. The model by Bepler and Berger [2019] consists of two parts. The first one is a protein language model, pretrained on all protein domain sequences of the Pfam database (21,827,419 sequences vs. ESM-2: around 65 million unique sequences). The model predicts a masked amino acid from context. The architecture is a 2-layer bidirectional LSTM (1024 units each), followed by a final 21-dimensional layer for amino acid prediction. After pretraining, the model weights are frozen. The second part of the embedding model is a 3-layer bidirectional LSTM (2 times 1024 units each), followed by a final 100-dimensional layer. For the final embedding, the hidden layers as well as the 21-dimensional layer are concatenated, yielding a 6165-dimensional protein embedding.

During training, an amino acid sequence is first encoded by the pretrained model. The hidden states are extracted, concatenated with the one-hot encoded amino acid sequence, and projected to a 512-d vector. This vector is the input for the second model. The training loss is a combination of a similarity loss and a contact loss. The similarity loss computes a structural similarity between pairs of proteins by first calculating a symmetric distance between their embeddings. From this score, an ordinal regression model predicts how much structural similarity two proteins share according to the SCOP hierarchy (no similarity, class similarity, fold similarity, etc.). The contact loss is obtained by feeding the embedding of a single protein through a small neural network which then predicts a within-protein contact matrix and computing the cross entropy.

The model was trained on the SCOPe ATRAL 2.06 dataset (22,408 sequences). For our experiment, we did not re-train the model but just embedded our sequences with the D-SCRIPT embed function which loads the weights provided by the authors.

One-hot encoding. For the one-hot encoding, we turn a sequence of length n into a matrix of size $n \times 24$ (every letter of the alphabet but J and O). Each column corresponds to one amino acid and contains 1 iff the amino acid is at position i, else 0. The Richoux model then zero-pads

the encodings to the maximum length occurring in the dataset such that all inputs have the same length.

1.2 Spectral normalization

In contrast to batch or layer normalization, spectral normalization is applied to the weight matrices instead of the output matrices. The idea is to divide the weight matrix by the spectral norm which is equal to the maximum singular value of the weight matrix:

$$W_{\text{SN}} = \frac{W}{\max_{h:h\neq 0} \frac{||Wh||_2}{||h||_2}}$$

Hence, the normalization controls how much the weight matrix can "stretch" any possible input. Like this, the Lipschitz constant is controlled because if values were close together in the input space, they cannot be stretched any further apart in the output space. This is because the spectral norm of the weight matrix is now equal to 1, which implies

$$\frac{||W_{\text{SN}}x - W_{\text{SN}}y||_2}{||x-y||_2} = \frac{||W_{\text{SN}}(x-y)||_2}{||x-y||_2} \leq \max_{h:h\neq 0} \frac{||W_{\text{SN}}h||_2}{||h||_2} = 1,$$

and hence

$$||W_{\text{SN}}x - W_{\text{SN}}y||_2 \leq ||x-y||_2,$$

for any input x, y with $x \neq y$. For more information, please refer to Miyato et al. [2018] or Liu et al. [2020].

1.3 Properties of the gold standard dataset

The gold standard dataset has originally been described in Bernett et al. [2024]. Shortly, we first partitioned the whole human proteome into three blocks where sequence similarity between the blocks was minimized. We then obtained positive PPIs from HIPPIE v2.3 and sorted them — according to the partition — into training, validation, and test set, such that a PPI is only part of the training set if both involved proteins are part of the first block of the partition and so on. Hence, no protein overlaps are present between the sets (Figure S1). We then randomly generated negative edges between the proteins occurring in each set such the expected number of negative edges incident with a a protein equals its node degree in the positive set (Figure S2). Hence, sequence similarity (Figure S3) and node degree biases are minimized.

For validating that sequence similarity was indeed minimized between the blocks, we built a custom BLAST database (command line tool, version 2.16.0+) and then queried all sequences against it (max_hsps 1). When bitscores of self-hits, as well as missing values, are set to 0, the mean bitscore of the training, validation, and test set were 0.630, 0.356, and 0.873 (without removing self-hits and setting NAs to zero: 96.49, 119.02, 113.80). Between training-test, training-validation, and validation-test, the mean bitscores were 0.023, 0.025, and 0.019 (without setting NAs to zero: 35.83, 39.46, 34.04). For the heatmap, proteins with less than 100 high-scoring pairs, as well as self-hits, have been filtered out for better readability.

2 Additional results: padding is unnecessary for TUnA

For the TUnA model, we also examined the impact of removing the padding from the input embeddings on the performance. As the TUnA model never uses any modules with trainable

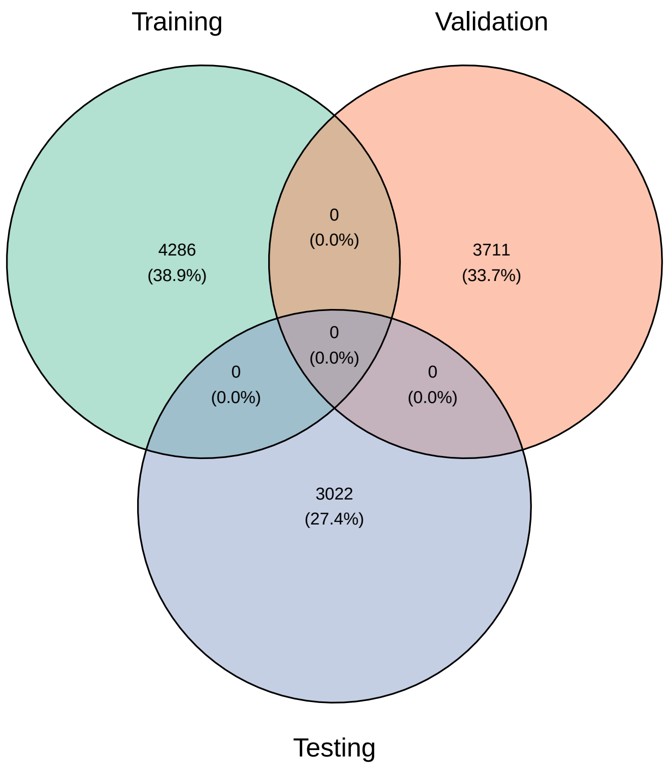

Figure S1: Venn diagram of the unique proteins occurring in their respective sets. There is no overlap between training, validation, and test set.

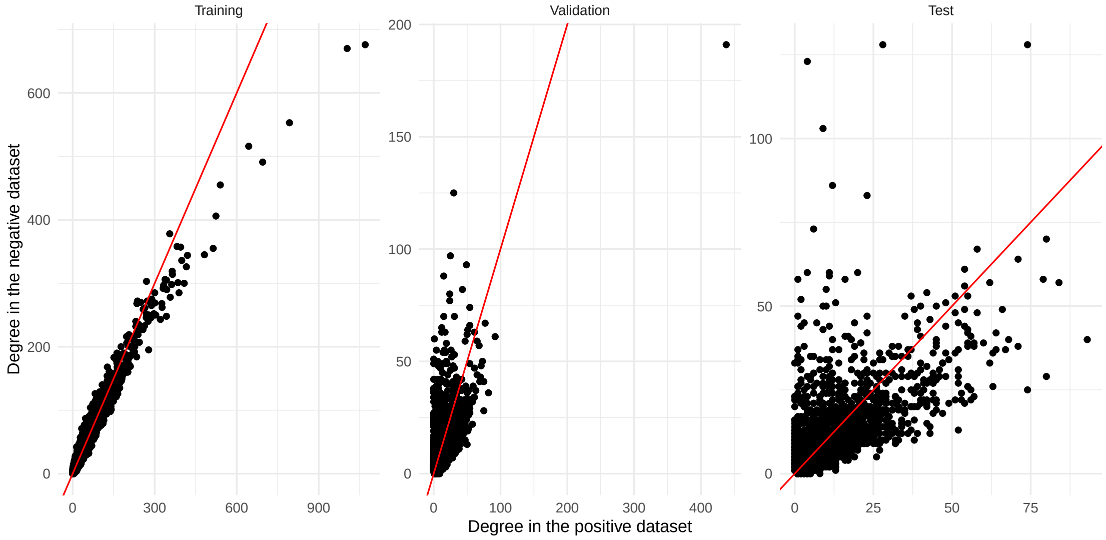

Figure S2: Individual protein degree in the positive vs. in the negative dataset. The node degrees are approximately preserved (the red line would be optimal).

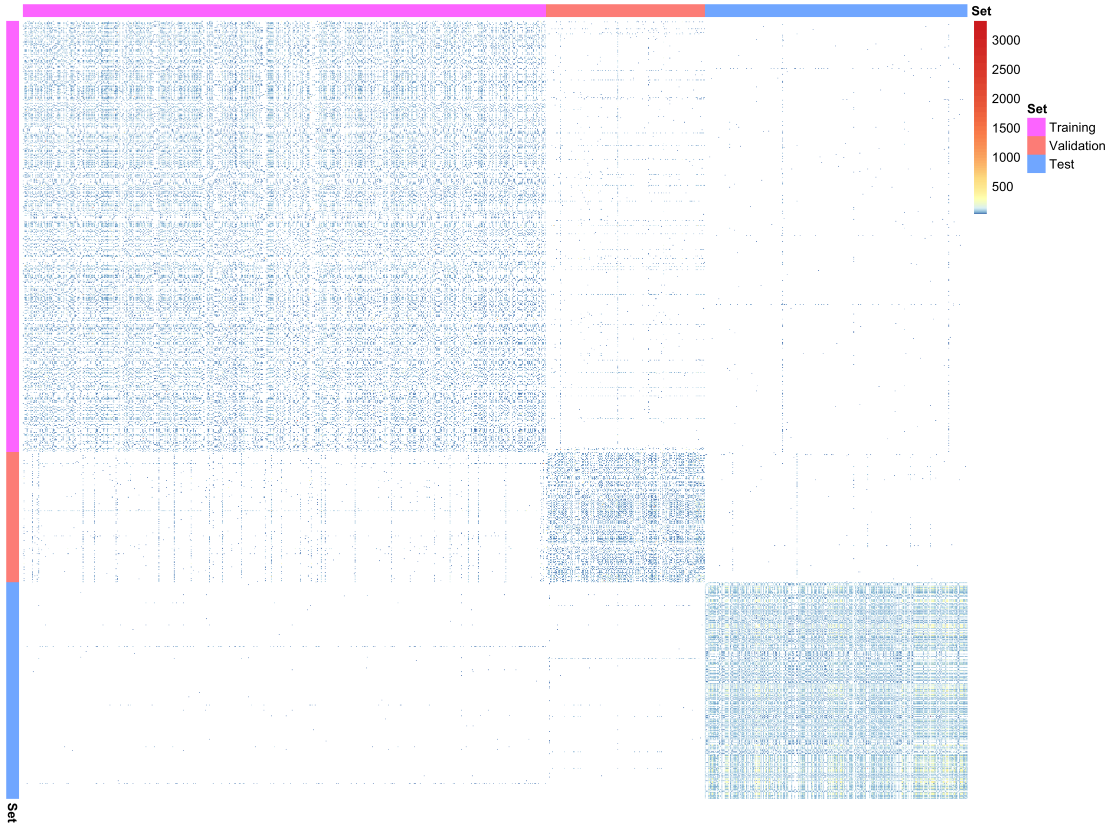

Figure S3: Heatmap of BLAST Bitscores between the proteins of training, validation, and test set. Proteins with more than 100 NA values were filtered for better readibility. Hence, especially the validation dataset seems smaller than it actually is because it has the lowest similarity within itself.

parameters to reduce the sequence dimension as well as mask padded positions in the attention mechanisms, we expected there to be no difference between using unpadded and padded inputs. This suspicion was confirmed by the test (Figure S13).

References

T. Bepler and B. Berger. Learning protein sequence embeddings using information from structure. *arXiv [cs.LG]*, 2019. doi: 10.48550/arXiv.1902.08661.

J. Bernett, D. B. Blumenthal, and M. List. Cracking the black box of deep sequence-based protein–protein interaction prediction. *Brief. Bioinform.*, 25(2):bbae076, 2024.

J. Liu, Z. Lin, S. Padhy, D. Tran, T. Bedrax Weiss, and B. Lakshminarayanan. Simple and principled uncertainty estimation with deterministic deep learning via distance awareness. In H. Larochelle, M. Ranzato, R. Hadsell, M. Balcan, and H. Lin, editors, *Advances in Neural Information Processing Systems*, volume 33, pages 7498–7512, 2020.

T. Miyato, T. Kataoka, M. Koyama, and Y. Yoshida. Spectral normalization for generative adversarial networks. *arXiv [cs.LG]*, 2018. doi: 10.48550/arXiv.1802.05957.

F. Richoux, C. Servantie, C. Borès, and S. Téletchéa. Comparing two deep learning sequence-based models for protein-protein interaction prediction. *arXiv [cs.LG]*, 2019. doi: 10.48550/arXiv.1901.06268.

S. Sledzieski, R. Singh, L. Cowen, and B. Berger. D-SCRIPT translates genome to phenome with sequence-based, structure-aware, genome-scale predictions of protein-protein interactions. *Cell Syst*, 12(10):969–982.e6, 2021.

3 Supplementary figures and tables

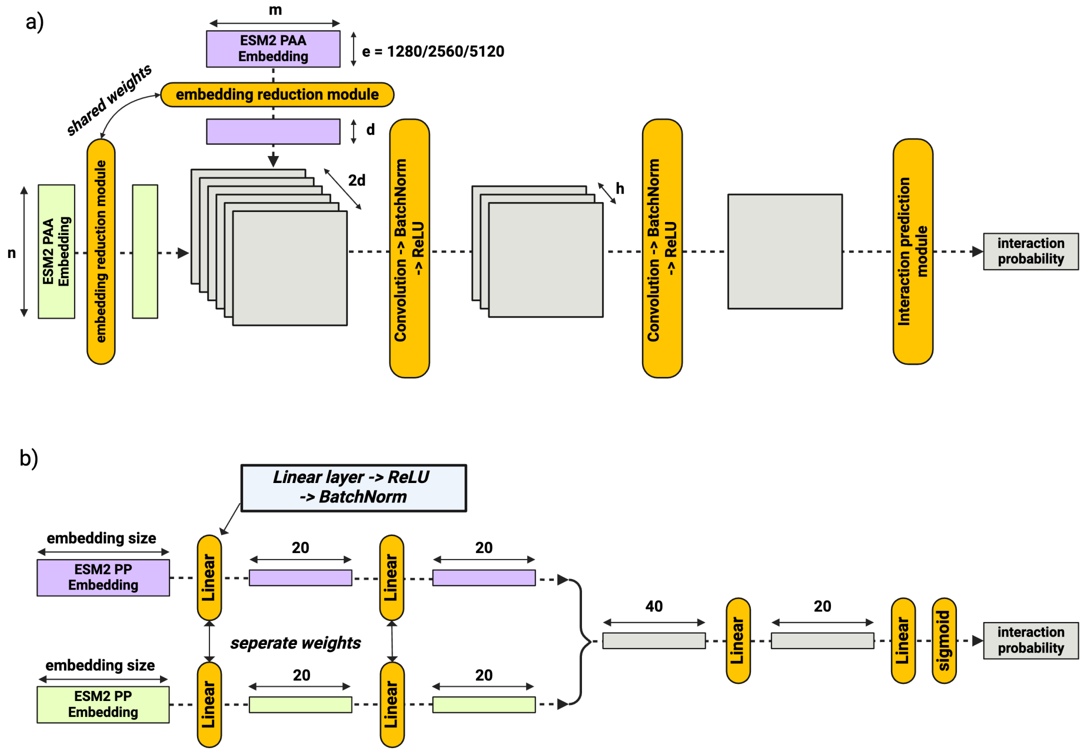

Figure S4: Visual representation of used Models. Rectangles represent the data, dimensions shown by arrows. Curved rectangles represent the modules used to change the data; a) D-SCRIPT-like; b) Richoux-like. Created in https://BioRender.com

Table S1: Overview and validation set performances of all tested configurations. PP: per-protein embedding, PT: per-token embedding.

Model	Accuracy	Model Class	Modification	Embedding Class
RFC-40 t33	0.56	RFC	Base	PP
RFC-40 t36	**0.58**	RFC	Larger Embedding	PP
RFC-40 t48	0.57	RFC	Larger Embedding	PP
RFC-400 t33	0.52	RFC	Base	PP
RFC-400 t36	0.52	RFC	Larger Embedding	PP
RFC-400 t48	0.52	RFC	Larger Embedding	PP
RFC-mean t33	0.53	RFC	Base	PP
RFC-mean t36	0.53	RFC	Larger Embedding	PP
RFC-mean t48	0.52	RFC	Larger Embedding	PP
2d-baseline t33	**0.57**	2d-baseline	Base	PT
2d-baseline t36	0.53	2d-baseline	Larger Embedding	PT
2d-baseline t48	0.52	2d-baseline	Larger Embedding	PT

2d-Selfattention t33	**0.60**	2d-Selfattention	Base	PT
2d-Selfattention t36	0.57	2d-Selfattention	Larger Embedding	PT
2d-Selfattention t48	0.54	2d-Selfattention	Larger Embedding	PT
2d-Selfattention-no-spectral	0.50	2d-Selfattention	No Spec. Norm	PT
2d-Selfattention-encoder-pre-reduction	0.56	2d-Selfattention	Encoder pre reduction	PT
2d-Crossattention t33	**0.62**	2d-Crossattention	Base	PT
2d-Crossattention t36	0.59	2d-Crossattention	Larger Embedding	PT
2d-Crossattention t48	0.59	2d-Crossattention	Larger Embedding	PT
2d-Crossattention-encoder-pre-reduction	0.58	2d-Crossattention	Encoder pre reduction	PT
2d-Crossattention-no-spectral	0.50	2d-Crossattention	No Spec. Norm	PT
Richoux-ESM-2 t33	0.63	Richoux-like	Base	PP
Richoux-ESM-2 t36	**0.64**	Richoux-like	Larger Embedding	PP
Richoux-ESM-2 t48	0.63	Richoux-like	Larger Embedding	PP
Richoux-ESM-2-spectral	0.63	Richoux-like	Spec. Norm	PP
Richoux-ESM-2-encoder-no-spectral	0.50	Richoux-like	No Spec. Norm	PP
Richoux-ESM-2-encoder-spectral	0.58	Richoux-like	Encoder pre reduction	PP
D-SCRIPT-ESM-2 t33	**0.63**	D-SCRIPT-like	Base	PT
D-SCRIPT-ESM-2 t36	0.62	D-SCRIPT-like	Larger Embedding	PT
D-SCRIPT-ESM-2 t48	0.56	D-SCRIPT-like	Larger Embedding	PT
D-SCRIPT-ESM-2-encoder-pre-reduction	0.51	D-SCRIPT-like	Encoder pre reduction	PT
D-SCRIPT-ESM-2-encoder-pre-reduction-no-spectral	0.50	D-SCRIPT-like	No Spec. Norm	PT
D-SCRIPT-ESM-2-encoder-post-reduction	0.62	D-SCRIPT-like	Encoder post reduction	PT
D-SCRIPT-ESM-2-encoder-crossattention	0.62	D-SCRIPT-like	Cross-attention	PT
TUnA t33	0.64	TUnA-like	Base	PT
TUnA t36	0.62	TUnA-like	Larger Embedding	PT
TUnA t48	0.61	TUnA-like	Larger Embedding	PT
TUnA-crossattention	**0.66**	TUnA-like	Cross-attention	PT
TUnA-unpadded	0.65	TUnA-like	No padding	PT
TUnA-no-spectral	0.50	TUnA-like	No Spec. Norm	PT

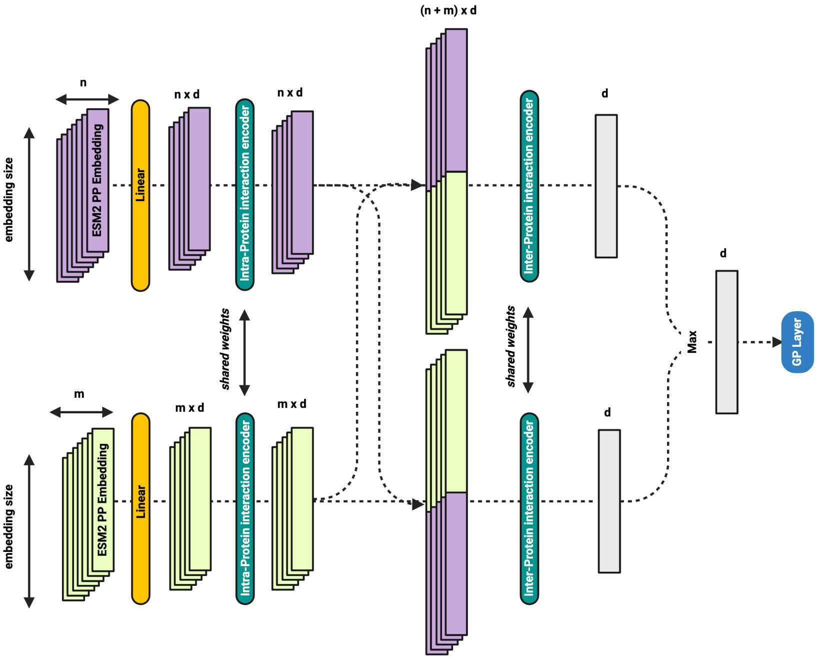

Figure S5: Visual representation of TUnA model architecture. Created in https://BioRender.com

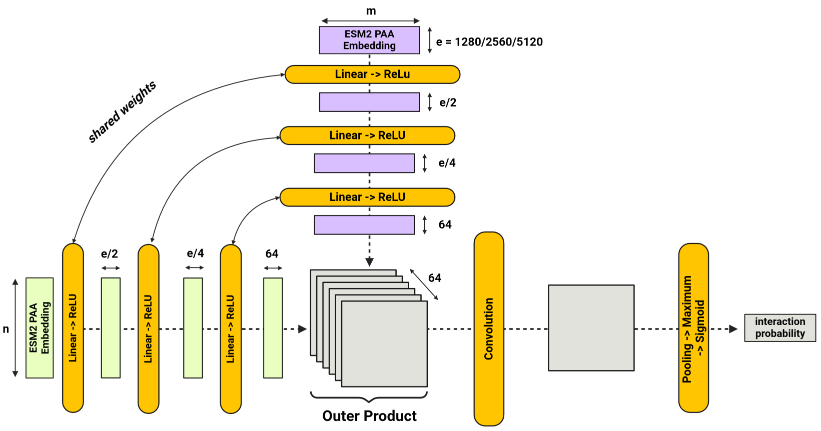

Figure S6: Visual representation of 2d-baseline. Rectangles represent the data, dimensions shown by arrows. Curved rectangles represent the modules used to change the data. Created in https://BioRender.com

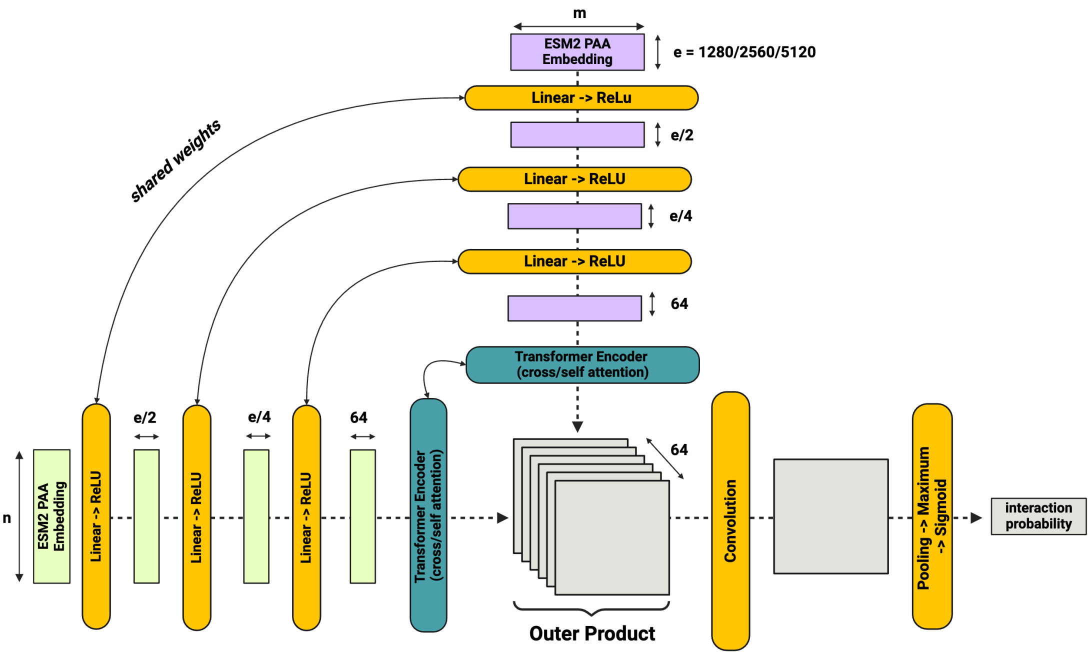

Figure S7: Visual representation of the attention models. Rectangles represent the data, dimensions shown by arrows. Curved rectangles represent the modules used to change the data. Created in https://BioRender.com

Table S2: Model accuracies on the gold-standard test set. Only the input was changed from ESM-2 embeddings to Bepler & Berger embeddings and one-hot encodings (architectures are the same as in Table 1). For Richoux, the one-hot encoding was padded as done in the original publication. PP: per-protein, PT: per-token embedding.

Model	ESM-2	Bepler & Berger	One-Hot	Embedding Type
RFC-40	0.577	–	–	PP
2d-baseline	**0.575**	0.569	0.501	PT
2d-Crossattention	**0.641**	0.613	0.502	PT
2d-Selfattention	**0.616**	0.581	0.501	PT
Richoux-ESM2	**0.633**	0.617	0.527	PP
D-SCRIPT-ESM-2	**0.628**	0.525	0.502	PT
TUnA	**0.645**	0.643	0.502	PT

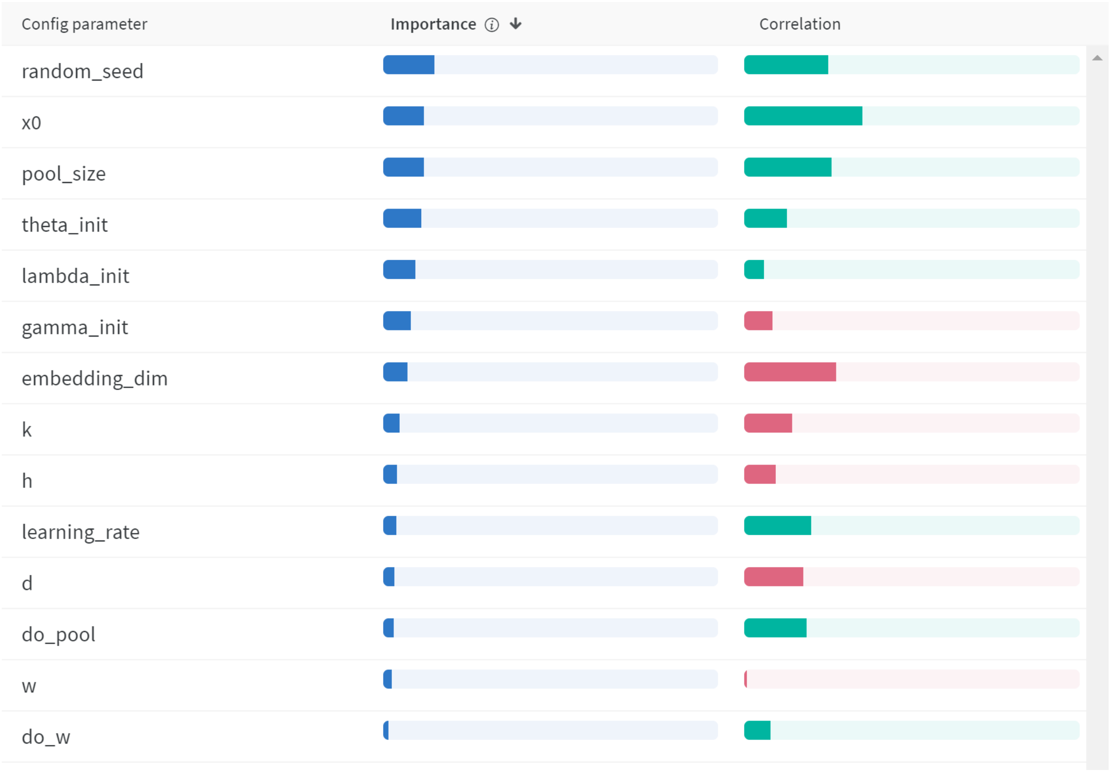

Figure S8: Importance and Pearson correlation of hyperparameters of the DSCRIPT-like model. The hyperparameter importance is a random forest feature importance calculated by Weights & Biases by fitting a random forest with the hyperparameters as input and the metric as output. "d" and "h" are used to reduce embedding size in the linear layer and convolution; "pool_size" and "w" control the kernel size of pooling and convolution, respectively; "theta_init" and "lambda_init" are used in the weighing of the contact map; "k", "gamma_init" and "x0" are parameters for the custom activation function; "do_pool" and "do_w" control whether the pooling and weighing will be performed. Created with wandb.

Table S3: Amount of filtered PPIs due to additional cofactors or ligands, homomers or short length; Interactions is the total number of entries in the PDB with exactly two distinct proteins that the models predicted confidently (> 0.9); Chains is the number of entries filtered out due to additional chains, i.e., cofactors, ligands or homomers; Length describes the number of entries filtered out due to at least one protein sequence being too short; Total Skipped is the total amount of skipped entries per model (does not account for duplicate entries); Percentage shows the percentage of filtered entries.

Model	Interactions	Chains	Length	Total Skipped	Percentage
DSCRIPT-like	21	13	5	18	86%
Crossattention	12	8	3	11	92%
Selfattention	32	18	7	25	78%
2d-baseline	19	15	4	19	100%
Total	84	54	19	73	87%

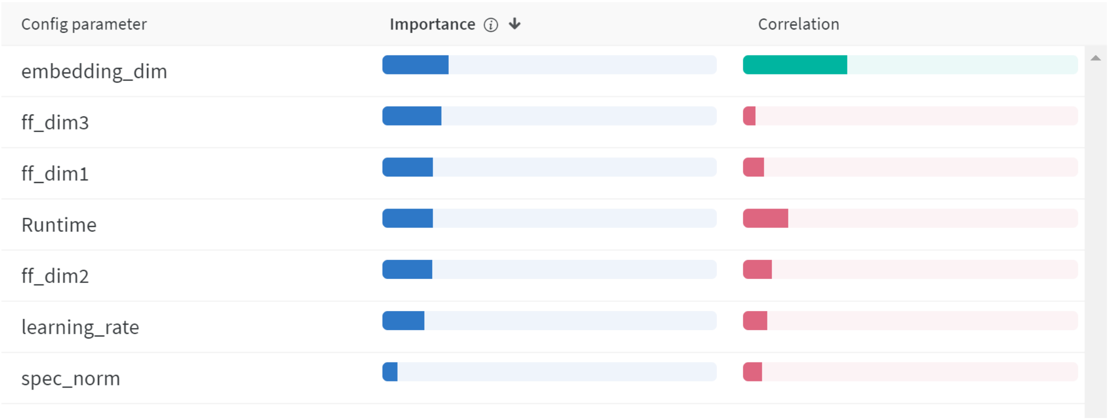

Figure S9: Importance and Correlation of hyperparameters of the Richoux-like model, Created with wandb.

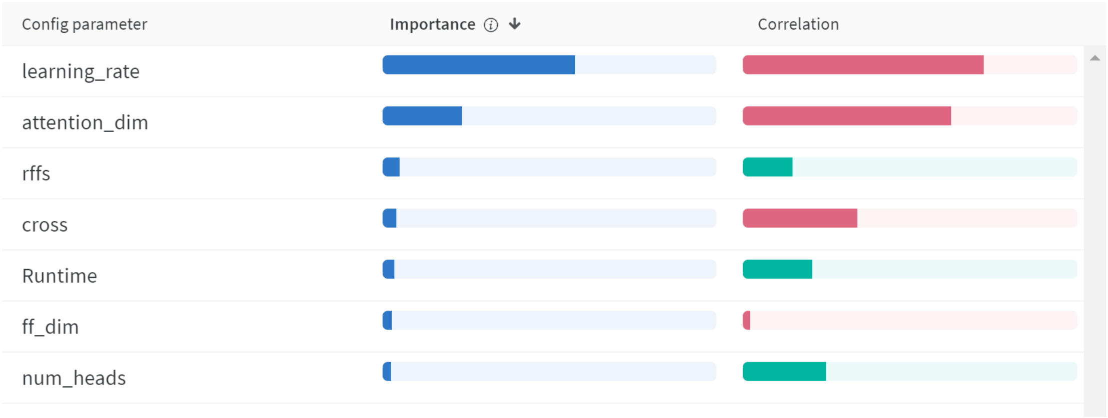

Figure S10: Importance and Correlation of hyperparameters of the TUnA model, Created with wandb.

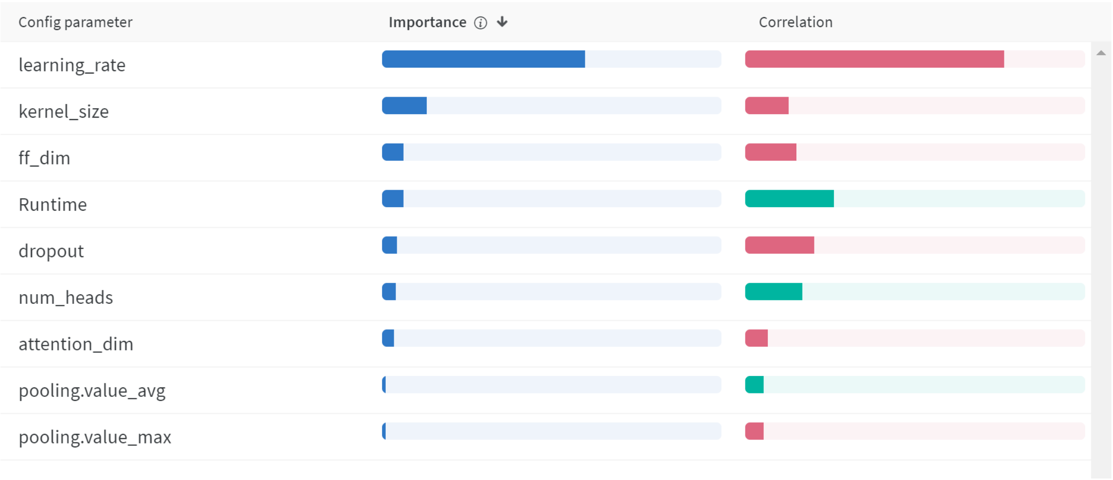

Figure S11: Importance and Correlation of hyperparameters of the Crossattention model, Created with wandb.

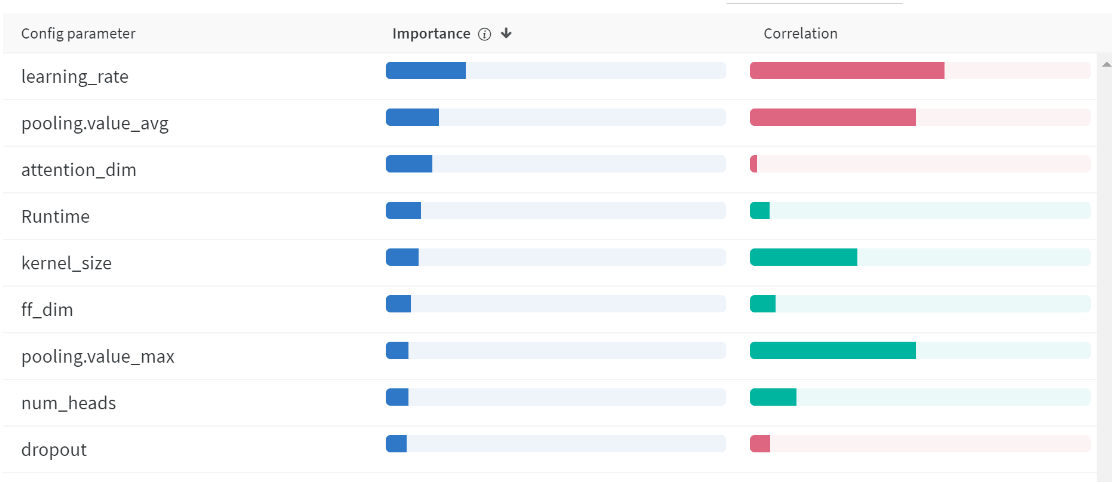

Figure S12: Importance and Correlation of hyperparameters of the Selfattention model, Created with wandb.

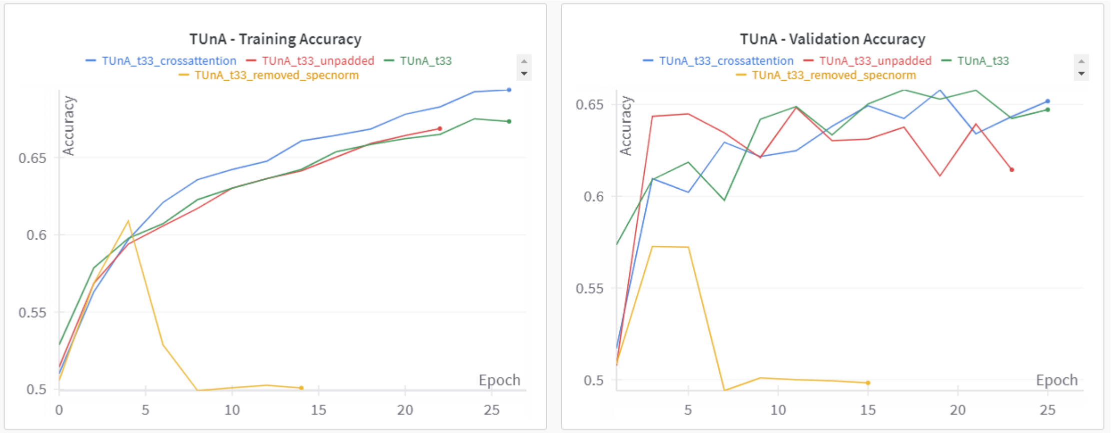

Figure S13: Comparison of TUnA models. Unmodified TUnA (green), TUnA with unpadded input (red), TUnA with cross attention in the encoders (blue), TUnA without spectral normalization (yellow). Created with wandb.

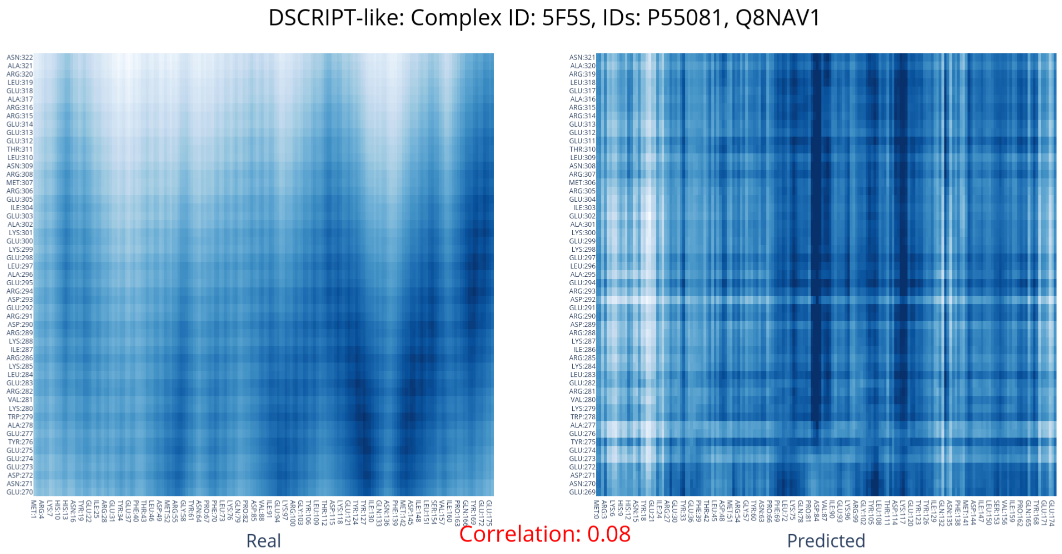

Figure S14: Comparison of the real and predicted distance map of the DSCRIPT-ESM-2 model for the 5F5S complex. White indicates low values (i.e., contact in the distance map), dark blue high values. The values of the implicitly predicted distance map correspond to the output of the final convolution layer of the D-SCRIPT-ESM-2 model followed by batch normalization and ReLU activation.

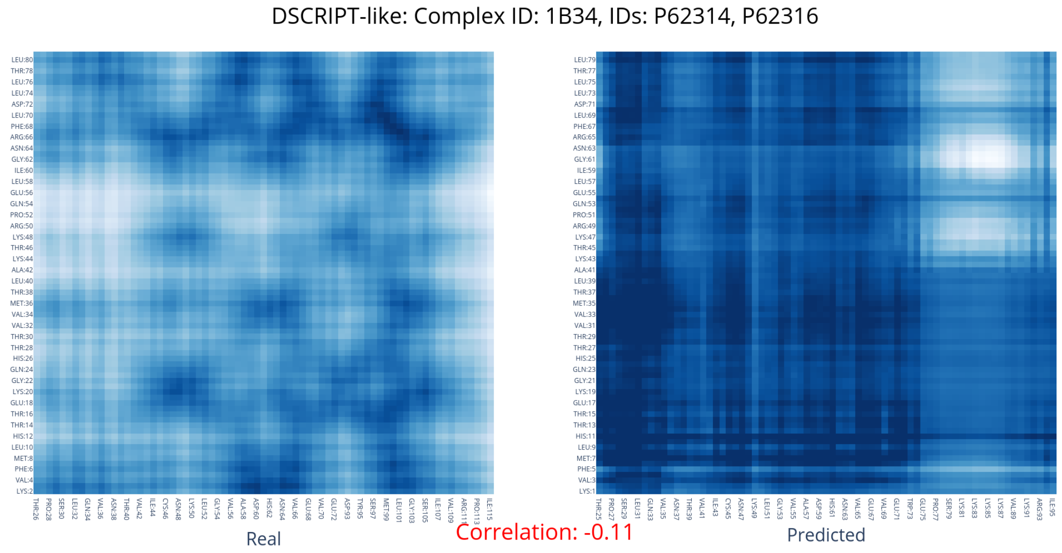

Figure S15: Comparison of the real and predicted distance map of the DSCRIPT-ESM-2 model for the 1B34 complex. White indicates low values (i.e., contact in the distance map), dark blue high values. The values of the implicitly predicted distance map correspond to the output of the final convolution layer of the D-SCRIPT-ESM-2 model followed by batch normalization and ReLU activation.

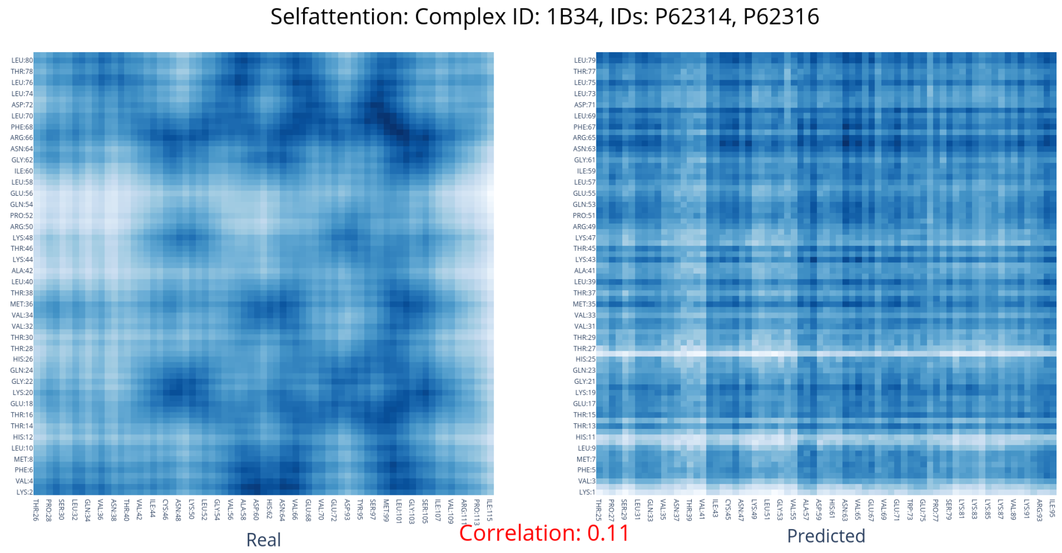

Figure S16: Comparison of the real and predicted distance map of the Selfattention model for the 1B34 complex. White indicates low values (i.e., contact in the distance map), dark blue high values. The values of the implicitly predicted distance map correspond to the output of the final convolution layer of the Selfattention model.

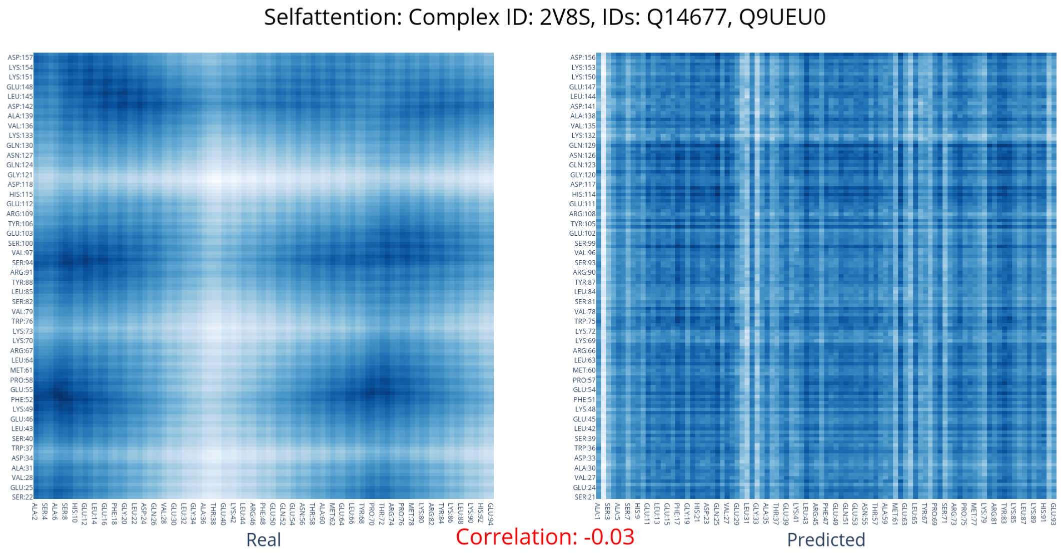

Figure S17: Comparison of the real and predicted distance map for the 2V8S: Selfattention model. White indicates low values (i.e., contact in the distance map), dark blue high values. The values of the implicitly predicted distance map correspond to the output of the final convolution layer of the Selfattention model.

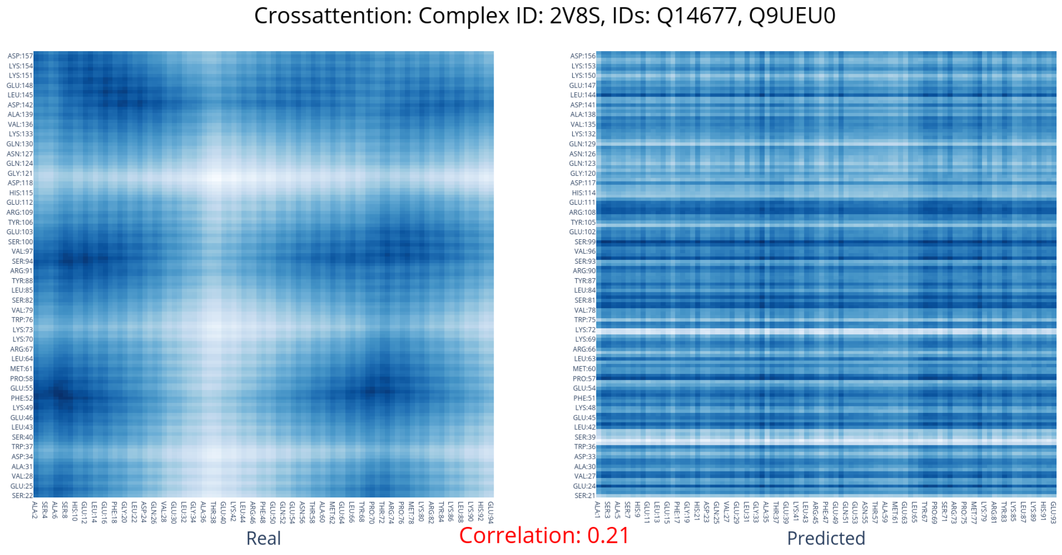

Figure S18: Comparison of the real and predicted distance map for the 2V8S: Crossattention model. White indicates low values (i.e., contact in the distance map), dark blue high values. The values of the implicitly predicted distance map correspond to the output of the final convolution layer of the Crossattention model.

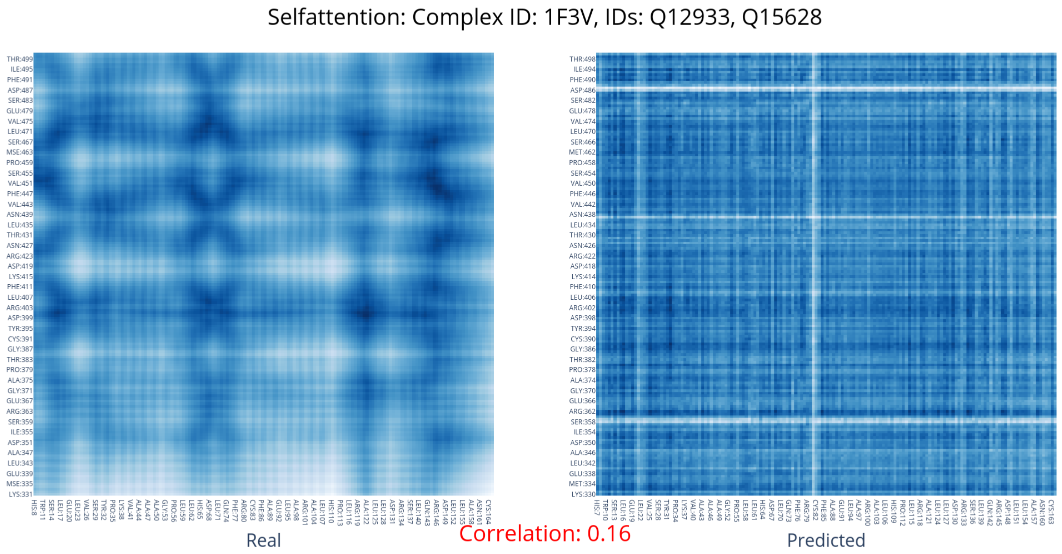

Figure S19: Comparison of the real and predicted distance map of the Selfattention model for the 1F3V complex. White indicates low values (i.e., contact in the distance map), dark blue high values. The values of the implicitly predicted distance map correspond to the output of the final convolution layer of the Selfattention model.

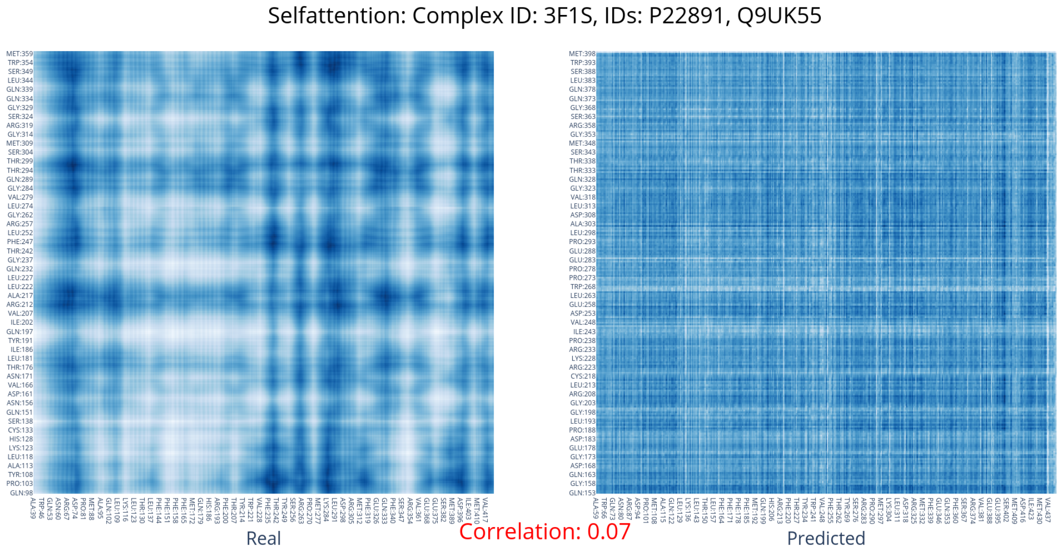

Figure S20: Comparison of the real and predicted distance map of the Selfattention model for the 3F1S complex. White indicates low values (i.e., contact in the distance map), dark blue high values. The values of the implicitly predicted distance map correspond to the output of the final convolution layer of the Selfattention model.

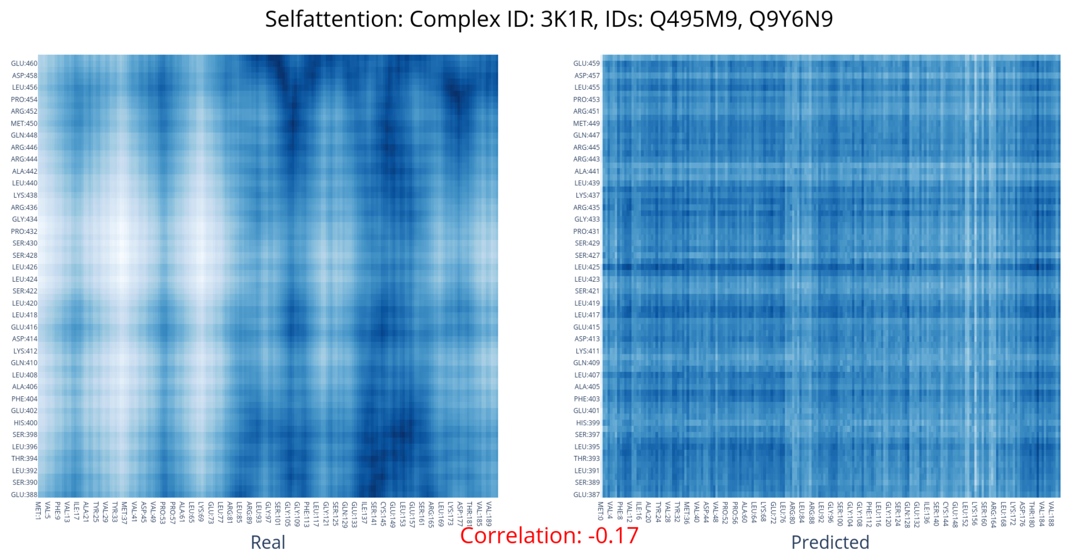

Figure S21: Comparison of the real and predicted distance map of the Selfattention model for the 3K1R complex. White indicates low values (i.e., contact in the distance map), dark blue high values. The values of the implicitly predicted distance map correspond to the output of the final convolution layer of the Selfattention model.

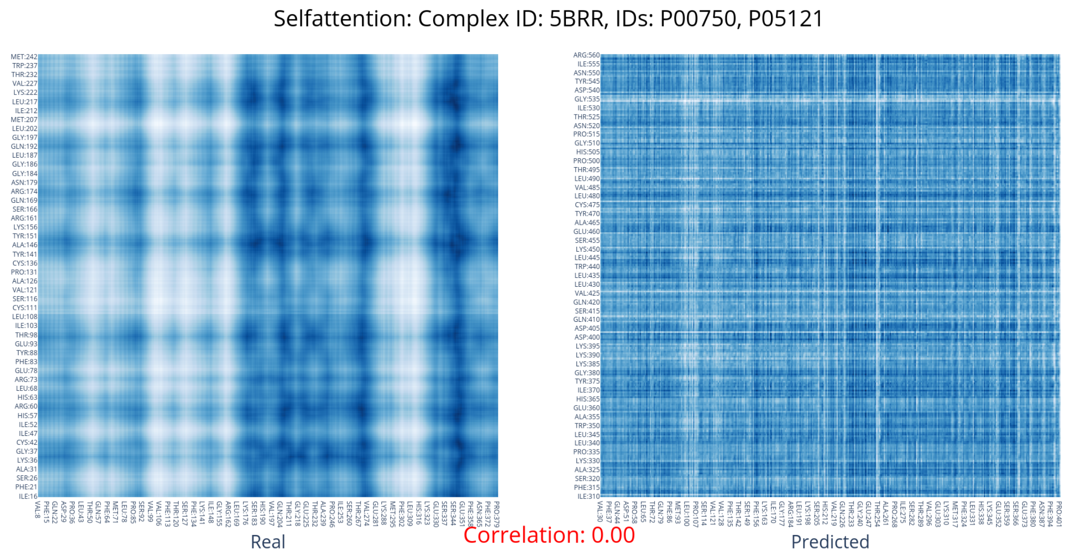

Figure S22: Comparison of the real and predicted distance map of the Selfattention model for the 5BRR complex. White indicates low values (i.e., contact in the distance map), dark blue high values. The values of the implicitly predicted distance map correspond to the output of the final convolution layer of the Selfattention model.

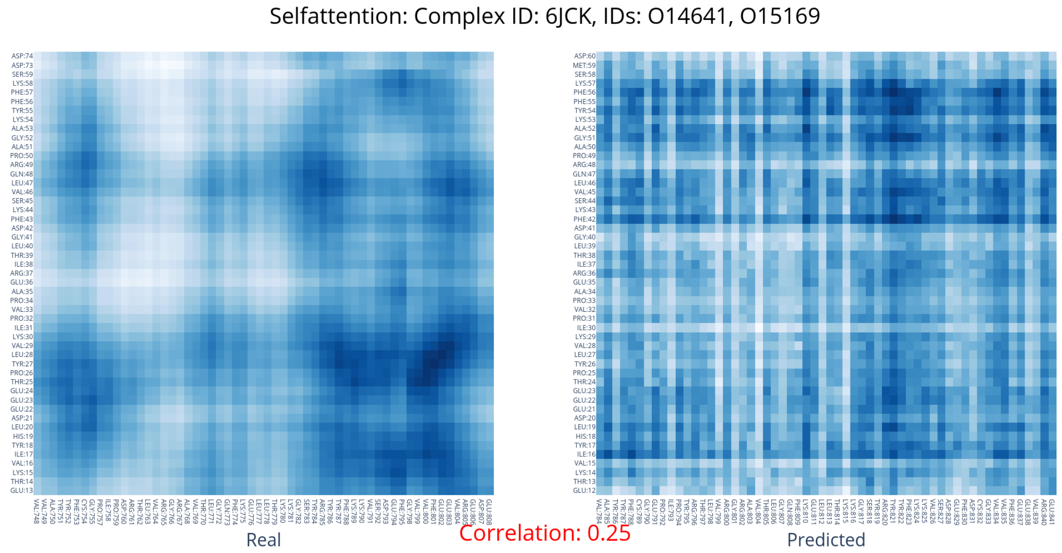

Figure S23: Comparison of the real and predicted distance map of the Selfattention model for the 6JCK complex. White indicates low values (i.e., contact in the distance map), dark blue high values. The values of the implicitly predicted distance map correspond to the output of the final convolution layer of the Selfattention model.

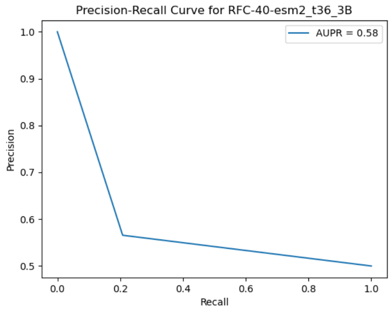

(a) Precision-recall curve RFC-40

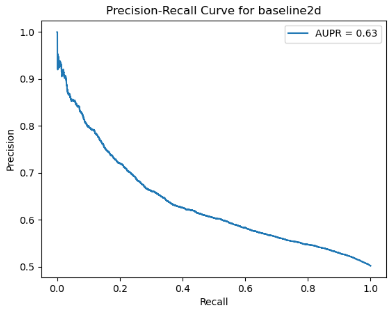

(b) Precision-recall curve 2d-baseline

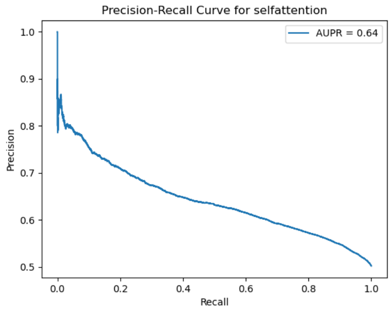

(c) Precision-recall curve Selfattention

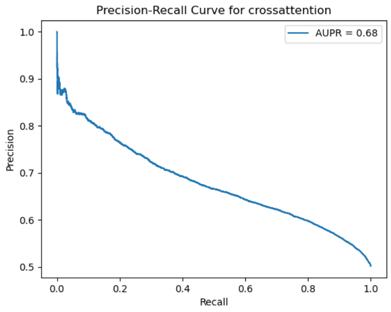

(d) Precision-recall curve Crossattention

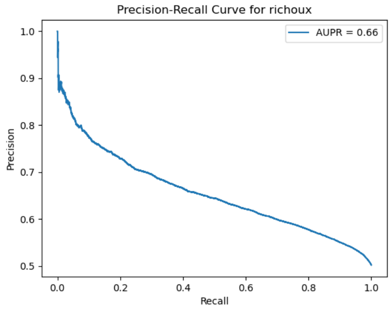

(e) Precision-recall curve Richoux-ESM2

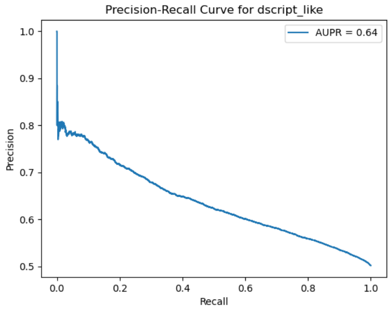

(f) Precision-recall curve D-SCRIPT-ESM2

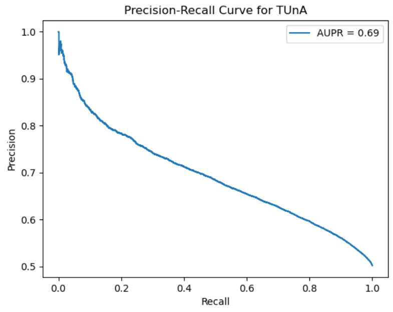

(g) Precision-recall curve TUnA

Figure S24: Precision-recall curves corresponding to the results in Table 1.
